# Improving Mechanical Properties of Starch-Based Hydrogels Using Double Network Strategy

**DOI:** 10.3390/polym14173552

**Published:** 2022-08-29

**Authors:** Jiradet Sringam, Porntipa Pankongadisak, Tatiya Trongsatitkul, Nitinat Suppakarn

**Affiliations:** 1 School of Polymer Engineering, Institute of Engineering, Suranaree University of Technology, Nakhon Ratchasima 30000, Thailand; 2 Research Center for Biocomposite Materials for Medical Industry and Agricultural and Food Industry, Suranaree University of Technology, Nakhon Ratchasima 30000, Thailand; 3 Center of Excellence in Biomechanics Medicine, Suranaree University of Technology, Nakhon Ratchasima 30000, Thailand; 4 Center of Excellence in Microbial Technology for Agricultural Industry, Suranaree University of Technology, Nakhon Ratchasima 30000, Thailand

**Keywords:** starch, poly(vinyl alcohol), single network hydrogel, double network hydrogel, cytotoxicity

## Abstract

This work aims to improve the mechanical properties of starch-based hydrogels using a double-network (DN) strategy. The single network (SN) starch hydrogel was first prepared using glutaraldehyde as a crosslinker. The compressive properties of the SN hydrogels were influenced by both crosslinker content and crosslinking time. The SN starch hydrogel possessing the best mechanical properties was then fabricated into DN hydrogels. Poly(vinyl alcohol) (PVA) and borax were used as a secondary polymer and a crosslinker, respectively. The PVA–borax complexation partly enhanced the DN hydrogel’s compressive modulus by 30% and its toughness by 39%. DN hydrogels were found to have denser microstructures than SN hydrogels. To be specific, their walls thickened and grew more continuous while their pores shrank. The increased crosslinking density resulted in changes to the microstructure, which were well correlated with their porosity and water uptake capacity. An in vitro cytotoxicity test of the DN hydrogels revealed that they were non-toxic to chondrocytes. This work demonstrated that double networking is a simple but effective strategy for improving mechanical properties of starch-based hydrogels without sacrificing their biocompatibility. This approach can be used to tailor hydrogel properties to fulfill requirements for biomedical applications, such as tissue engineering and other related fields.

## 1. Introduction

Starch-based hydrogels have drawn substantially attention in the field of tissue engineering in the recent years [1]. Starch is recognized as an important biomaterial due to its non-toxicity, low cost, biocompatibility, predictable biodegradability, and immunogenicity [2]. Crosslinking of starch to form a three-dimensional network or hydrogel is possible because a starch molecule contains large number of hydroxyl groups that can easily be crosslinked [3,4].

In tissue engineering application, hydrogels of starch are attractive because they are functionally resemblant to natural tissue with a high degree of biocompatibility [5]. The soft texture and flexible nature of a hydrogel, similar to tissue characteristics, are attributed to its ability to retain a large amount of water, which is commonly found as a main solvent in biological fluids. As a result, a hydrogel can suppress an inflammatory response of the surrounding connective tissues [6,7]. The porous structure of a hydrogel, which resembles the extracellular matrix (ECM), is responsible for its inherent biocompatibility and also provides an encapsulation ability. A hydrogel can carry and protect the encapsulated therapeutic agents from degradation in the surrounding tissue environment. Thus, a hydrogel can be beneficially used as a carrier for growth factors or cells that assist tissue growth stimulation. However, the fragile nature of starch-based single network hydrogels (especially when swollen) limits their application ranges. For some applications, such as in the field of bone tissue engineering, hydrogels should possess strength and toughness that can withstand significant mechanical loads [8,9].

Numerous approaches have been used to develop a hydrogel with enhanced mechanical properties. Designing of a hydrogel by choosing different type and number of crosslinker were conventionally done. In the recent year, different strategies have been developed to create novel types of hydrogels. These include a hydrogel with a topological structure (TP), a hydrogel with a hybrid structure, a hydrogel with a nanocomposite structure (NC), and a hydrogel with double network structure (DN). Among these, making a double network hydrogel is a relatively new concept that has gained significant attention in the recent years. These DN hydrogels are formed by intertwining two different polymer networks together, resulting in a hydrogel with altered properties that are contributed by both polymer networks. Various properties of the DN hydrogels were reportedly improved over those of the SN hydrogels of their constituents [10]. For example, Gadhave et al. found that the DN hydrogels of maize starch and poly(vinyl alcohol) demonstrated nonlinearly enhanced mechanical properties as a result of the combination of rigid polysaccharide chains and ductile PVA chain [11]. Dixit et al. also reported success in improving hydrogel mechanical properties using a DN strategy. In their work, they prepared double network hydrogels using PVA and poly(acrylamide-*co*-2-hydroxyethyl methacrylate) or P(AM-HEMA) as the primary and secondary polymers, respectively. Borax and *N*,*N*′-methylenebisacrylamide were used as crosslinkers for the primary and secondary polymers, respectively. The resulting DN hydrogels were strong and stretchable. The improvement in tensile strength to 60% higher than that of the single network PVA hydrogel was due to the complex formed by PVA–borax on the P(AM-*co*-HEMA) network [12].

Choosing the second network is crucial in order to improve mechanical properties of the fragile starch-based hydrogels [13]. Synthetic polymers are thought to be good candidates. This is because there are vast variety of the synthetic polymers, and their properties can easily be controlled and optimized [14]. For tissue engineering applications, several synthetic hydrogels have been prepared and extensively investigated, including poly (2-hydroxyethyl methacrylate) (P-HEMA), polyethylene glycol (PEG), polyacrylamide (PAM), and poly(vinyl alcohol) (PVA) [15]. Among these synthetic polymers, poly(vinyl alcohol) (PVA) is of our interest. PVA is a non-toxic, biodegradable polymer. Each PVA repeating unit has a hydroxyl group that allows PVA to crosslink chemically or physically in different hydrogel systems, hence expanding its potential applications.

Crosslinking agents for PVA include ethylene glycol diamine methacrylate (EGDMA) [16], bis(hydroxyethyl) sulfone (BHES) [17], and borax [12]. Among these, PVA-borax hydrogel system has received considerable attentions. One of the reasons is that PVA-borax hydrogel possesses malleability and ductility which can be a remedy for a fragile/brittle starch hydrogel [18]. In PVA-borax hydrogel, the network is formed by the formation of a “di-ol” complex between one borate ion and two di-ol units [12,19]. The resulting hydrogels have been proposed to be used as a cartilage substitute and to repair meniscus defects [20] as they possess an extremely low friction coefficient and minimum wear when rubbed against artificial joint materials [21].

In this study, we focused on improving the mechanical properties of starch-based hydrogels using the DN strategy with the ultimate goal of using the hydrogel in bone tissue engineering applications. To be specific, hydrogels are being developed to be used as an articular cartilage in the superficial zone, which requires a compressive modulus of the hydrogel in the range of 20–1160 kPa [22]. Therefore, compressive modulus is one of the key criteria to determine the suitability of the hydrogels for the applications. In our work, SN starch hydrogels were first prepared using glutaraldehyde (GA) as a crosslinker. The native cassava starch was chosen to prepare a single network hydrogel in this study as it was abundantly available [23]. Furthermore, understanding the nature and properties of the hydrogel made of a native starch would provide a baseline for other starch hydrogels, specifically modified starches, where the properties could be expectably improved. GA was chosen as it was known to be highly reactive and low cost. It acted as multifunctional crosslinker that formed bonds with the hydroxyl groups of starch molecules [24]. An investigation of the effects of varying GA content and crosslinking time on their morphology, porosity, water uptake capacity, and compressive properties was carried out. The SN starch hydrogel with the highest mechanical properties was then chosen and used to fabricate DN hydrogels [25]. PVA was used as a secondary polymer. Since PVA is a water-soluble polymer, it can be successfully interpenetrated into the starch hydrogel structure. Meanwhile, borax was used as a crosslinker. Moreover, the effects of varying borax concentration on the properties of DN hydrogel were investigated. Chemical structures, morphologies, porosity, water uptake, and mechanical properties were observed and measured. Additionally, in vitro cytotoxicity measurements were performed, and the results were compared between SN and DN starch hydrogels. Systematic studies of these parameters would provide the knowledge necessary for designing novel materials suitable for tissue engineering applications.

## 2. Materials and Methods

### 2.1. Materials

Native cassava starch was purchased from Kriangkrai Co., Ltd. (Nakornprathom, Thailand). GA (25 wt% in aqueous solution) was purchased from Thermo Fisher Scientific (Waltham, MA, USA). PVA (Mw ≈ 100,000 g/mol with 86–90% degree of hydrolysis) was purchased from Chem-Supply Pty., Ltd. (Gillman, Australia). Sodium tetraborate decahydrate (borax) was purchased from QRëC^TM^ (Auckland, New Zealand). Deionized water was used in this study.

### 2.2. Methods

#### 2.2.1. Preparations

Preparation of single network (SN) starch hydrogels

To prepare a SN Starch hydrogel, a 10 w/v% starch solution was gelatinized at 90 °C and mechanically stirred for 30 min until a transparent starch solution was obtained. Then, 50 mL of 2.5 M GA solution was added to the gelatinized starch solution and stirred for 2 min. The mixture was poured into round disk molds with a diameter and a thickness of 15.0 cm and 1.5 cm, respectively. Then the filled molds were placed in an oven at 60 °C for different crosslinking times: 2, 4, 6, 8, 10, and 24 h. After a predetermined crosslinking time was reached, the starch hydrogel samples were removed from the molds and left to dry at room temperature for 3 days, until they reached a constant weight. The samples were kept in a dry place at room temperature before test and characterization.

To prepare SN starch hydrogels with varying GA concentrations, GA stock solution with 2.5 M was used. GA working solutions were prepared from the stock solution at different amount, i.e., 10, 20, 30, 40, and 50 mL and DI water was added to the solutions to make a constant final volume of 50 mL. The hydrogels with various concentrations of GA were designated as SN10, SN20, SN30, SN40, and SN50, respectively.

Preparation of double network (DN) starch hydrogels

**Scheme 1:**
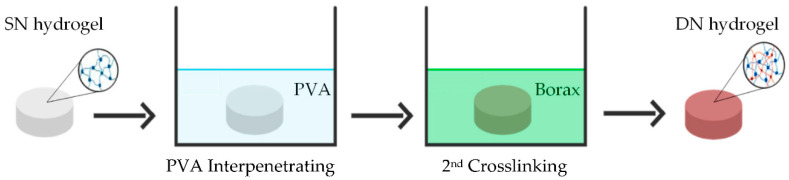
Schematic of DN hydrogel preparation. A DN hydrogel was prepared by interpenetrating the SN starch hydrogel with PVA and then crosslinking with borax at room temperature.

SN starch hydrogel possessing the highest compressive modulus and strength was used to fabricate into DN hydrogels. Borax was used as a secondary crosslinker to form a 3D network of PVA with the primary starch network. To prepare a DN hydrogel, the dried SN starch hydrogel was immersed in 50 mL of 3 wt% PVA for 24 h at room temperature. The product of this stage was called an interpenetrating network (IPN) hydrogel. This immersion method of DN hydrogel preparation was adapted from Dixit et al. [12]. After immersion, the IPN starch hydrogel was dried at 60 °C for 24 h. Then, it was immersed in a 50 mL of borax solution for 24 h to form DN hydrogels. The DN hydrogels were prepared with different borax concentrations of 0.05, 0.10, and 0.15 M and designated as DN0.05, DN0.10, and DN0.15, respectively. The procedure for preparing DN hydrogel is illustrated in Scheme 1.

#### 2.2.2. Chemical Structure Analysis

Chemical functional groups of SN and DN starch hydrogels were identified by a Bruker Tensor 27 Fourier transform infrared (FTIR) spectrometer (Bruker, Billerica, MA, USA). Dried samples were ground with analytical grade KBr and pressed into transparent disks prior to the measurements. Their spectra were recorded over a wavenumber range of 4000 to 400 cm^−1^ with a resolution of 4 cm^−1^ and a number of scans of 64 [26].

#### 2.2.3. Morphology

The morphologies and porous structures of hydrogels were investigated by a FEI Quanta 450 Scanning electron microscopy (SEM) (Philips, Hillsboro, OR, USA). Before characterization, hydrogel samples were frozen at −60 °C for 24 h before being lyophilized for 48 h in a Gamma 1-16 LSCplus freeze dryer (Martin Christ, Osterode am Harz, Germany). The dried hydrogel cross-sections were then sputtered with a thin layer of gold. This SEM characterization method was adapted from the work of Podhorská et al. [27]. The pore size and wall thickness of dried hydrogels were measured using ImageJ, image analyzer software. In addition, energy dispersive spectroscopy (EDS) was used to detect and quantify Boron (borax crosslinker) on the hydrogels’ surfaces.

#### 2.2.4. Porosity

The porosity of a hydrogel sample can be quantified by measuring the amount of ethanol that penetrates the sample. Since ethanol is non-solvent for starch hydrogel, it simply filled the pores and the free space in the network structure of the hydrogel. The porosity of hydrogels was measured using the solvent replacement method. The hydrogel discs with known weight (W_1_) were immersed in pure ethanol for 24 h. After that, the hydrogels were removed from the ethanol, blotted with tissue paper before being weighed again (W_2_). The mass of hydrogels was weighed by a ML204/01 analytical balance (Mettler Toledo, Greifensee, Switzerland). The percentage of porosity was calculated using the following Equation (1):

(1) $$\mathrm{Porosity}\;\left(\%\right)=\frac{W_{2}-W_{1}}{\rho V}\;\times \;100$$

where W_1_ and W_2_ are the weights of a hydrogel before and after immersing in absolute ethanol, respectively. ρ is the density of ethanol and V the volume of a hydrogel [28].

#### 2.2.5. Water Uptake Capacity

Water uptake of a hydrogel is the total amount of the water absorbed by the material. The water resides in the pore and between polymer chains. Due to the microporous structure and hydrophilic nature of the hydrogels, water can enter both the pores and the free space in the network structure of the pore wall. Therefore, water uptake by a hydrogel is typically used as an indicator for the degree of crosslinking. Water uptake capacity of hydrogels was measured by soaking the dried hydrogel samples in distilled water until they reached an equilibrium swelling weight (W_s_) (about 24 h). After that, the hydrogels were dried at 60 °C in a hot air oven and reweighed (W_d_) [12]. The mass of hydrogels was weighed by a ML204/01 analytical balance (Mettler Toledo, Greifensee, Switzerland). The percentage of water uptake was calculated using Equation (2).

(2) $$\mathrm{Water}\;\mathrm{uptake}\;\left(\%\right)=\frac{W_{s}-W_{d}}{W_{d}}\;\times \;100$$

#### 2.2.6. Compressive Properties

Compressive properties of hydrogels were determined using a TA.XT Plus Texture analyzer (Stable Micro Systems Ltd., Surrey, UK). A fully swollen hydrogel sample was cut into a cylindrical disk with a diameter of 11 mm and a thickness of 2 mm. Compressive tests were performed using a 1 kgF load cell at a strain rate of 0.05 mm/s. The testing method was adapted from Llorens-Gámez et al. [29]. Five replicates of hydrogel specimens were tested for each experimental condition. The compressive modulus was determined using the slope of the initial linear portion (5–10% strain) of the stress–strain curves. The compressive strength was the maximum compressive stress at the breaking point. The toughness was calculated from the area under the stress–strain curve using the Origin^®^ software (OriginLab Corporation, Northampton, MA, USA).

#### 2.2.7. In Vitro Cytotoxicity

A preliminary study on the cytotoxicity of the prepared hydrogels was conducted in accordance with ISO standard 10993-5: Biological evaluation of medical devices. As cultivated cells, primary human chondrocytes (HC) from articular cartilage (collected and approved by the Ramathibodi Hospital, Thailand) were employed. Dulbecco’s Modified Eagle Medium (DMEM: Gibco, Billings, MT, USA) with 1% penicillin-streptomycin, 1% L-glutamine, and supplemented with 10% fetal bovine serum (FBS: Gibco, Billings, MT, USA) was used as a culture medium. Prior to starting the assays, the HC cells were grown to 80% culture confluence in 75 cm^2^ cell culture flasks (SPL Life Sciences Co., Ltd., Seoul, Korea) under standard culture conditions of 37 °C and 5% CO_2_.

The tests were conducted on the extracts of culture media in serum-free medium (SFM) using a similar approach as Pankongadisak et al. [30]. The hydrogel sample was first immersed in SFM for 24 h at 37 °C. The extract medium was then diluted with SFM to obtain the four concentrations (i.e., 0.5, 5, 10, and 50 mg/mL). Before testing, the extract media were sterilized with 0.22 µm of a Minisart^®^ syringe filters (Sartorius Lab Instruments GmbH & Co. KG, Goettingen, Germany).

Cells were counted using the Trypan blue (Sigma-Aldrich^®^, St. Louis, MO, USA). The cells were plated at a seeding density of 5 × 10^4^ viable cells/cm^2^ in SPL 96-well culture plates (flat bottom) using a multichannel pipette. After an incubation period of 24 h at 37 °C with 5% CO_2_, the culture medium was removed and replaced with the extract media. Cells were then incubated for 24 h at 37 °C and 5% CO_2_ before being tested for viability using the 3-(4,5-dimethylthiazol-2-yl)-2,5-diphenyltetrazolium bromide (MTT) assay. The viability of cells cultured by the fresh SFM was used as the control. After the treatment, the medium was discarded, and the samples were washed with PBS. 100 µL of MTT solution (0.5 mg/mL) was added and incubated for 3 h. After decanting the MTT solution, 100 µL of dimethyl sulfoxide was added to dissolve the formazan crystals. After 2 min of agitation, the solutions’ absorbance at 570 nm was determined using a Multiskan^TM^ GO Microplate spectrophotometer (Thermo Scientific, Waltham, MA, USA). Cell viability was calculated according to the following Equation (3):

(3) $$v\;(\%)=\frac{{100\;\times \;\mathrm{OD}}_{570A}}{{\mathrm{OD}}_{570B}}$$

where v denotes cell viability, OD_570A_ is a value of the measured optical density of the treated cells, and OD_570B_ is a value of the measured optical density of the non-treated cells.

### 2.3. Statistical Analysis

Statistical analysis was performed using the IBM SPSS Statistic, version 24.0 (IMB corp., Armonk, NY, USA). The means and standard deviation of each result were displayed. The compressive test was performed on five replicate samples (*n* = 5), whereas the other three tests (porosity, water uptake, and cell cytotoxicity) were conducted on three replicate samples (*n* = 3). All quantitative data were analyzed using one-way ANOVA and Turkey’s post hoc comparison test. To identify statistical differences between the comparison groups, a value of *p* < 0.05 was employed as the level of statistical significance.

## 3. Results and Discussion

### 3.1. Chemical Structure Analysis of Starch Hydrogels

**Figure 1:**
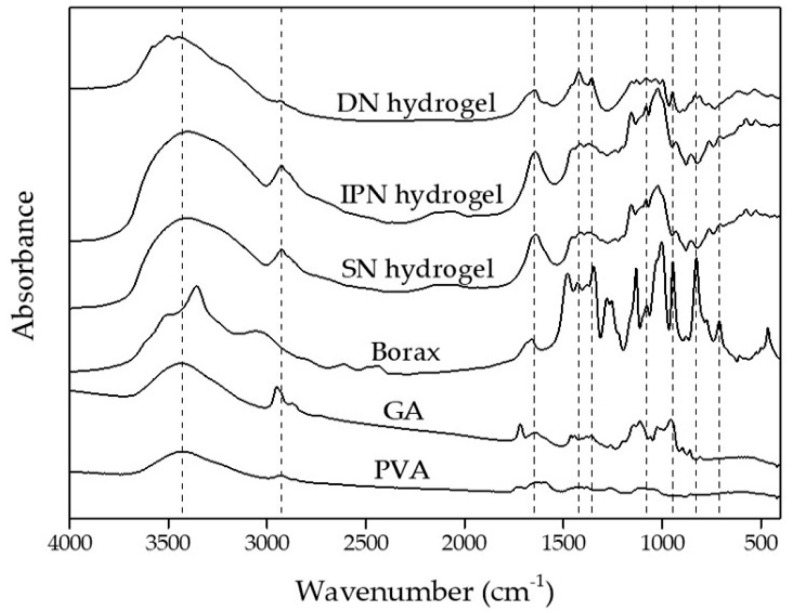
FTIR spectra of PVA, GA, Borax, and SN, IPN, and DN starch hydrogels.

FTIR spectra of PVA, GA, Borax, SN, IPN and DN starch hydrogels are shown in Figure 1. The SN starch hydrogel spectrum presented absorption bands at 3431, 2928, and 1643 cm^−1^ which related to O-H stretching, C-H stretching, and C-O bending of OH groups, respectively [31]. The C=O stretching at 1712 cm^−1^, as shown in GA spectrum was aldehyde functional groups of GA. This peak was absent from the spectrum of the SN starch hydrogel. Instead, a new peak at around 1063 cm^−1^ was observed and assigned to the acetal bond. This suggested that crosslinks between GA and starch occurred via the formation of acetal bridges between GA and the hydroxyl groups of starch [32]. For the IPN hydrogel, the absorption band of hydroxyl groups appeared in the same range as those of the SN and DN starch hydrogels [33,34]. Nonetheless, the intensity ratio between O-H stretching and C-H stretching in IPN hydrogel differed from that in DN hydrogel. The spectrum of the DN hydrogel revealed several distinct peaks for the borax and borate ions, including B-O-C bonds at 1421 and 1355 cm^−1^ (asymmetric stretching bands) and 947 cm^−1^ (symmetric stretching band), 661 cm^−1^ (bending of the B-O-B bond in the borate network), 833 cm^−1^ (B-O stretching from residual B(OH)_4_^−^) [18,35]. The new absorption peak at 1355 cm^−1^ (asymmetric stretching of B-O-C) was correlated to the hydroxyl groups that form complexes (crosslinks) with borate ions. These results suggested that the complexation of PVA and borate ions took place, which led to the formation of a physical crosslinking of the secondary network in the DN hydrogel.

### 3.2. Characterization of Single Network (SN) Starch Hydrogels

#### 3.2.1. Morphologies of SN Starch Hydrogels

**Figure 2:**
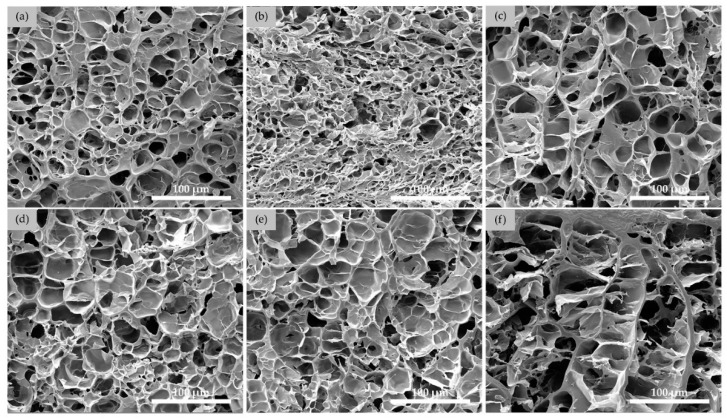
SEM micrographs of cross-sections of SN starch hydrogels containing 30 mL of GA content (SN30), at various crosslinking times (1000× of magnification): (**a**) 2 h, (**b**) 4 h, (**c**) 6 h, (**d**) 8 h, (**e**) 10 h and (**f**) 24 h.

**Figure 3:**
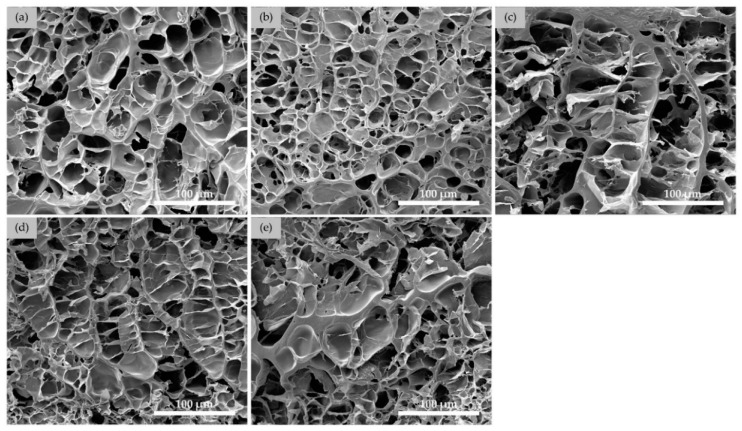
SEM micrographs of cross-sections of SN starch hydrogels after 24 h of crosslinking time, at various GA contents (1000× of magnification): (**a**) SN10, (**b**) SN20, (**c**) SN30, (**d**) SN40, and (**e**) SN50.

SEM micrographs of cross-sections of freeze-dried SN30 hydrogels, prepared at various crosslinking times and those of SN hydrogels, prepared at various GA contents are shown in Figure 2 and Figure 3, respectively. In general, all SN samples were porous with irregular pore and the closed-cell walls were interconnected. The macro porous structure possessed average pore size of >16 µm.

In Figure 2, SEM images of SN30 starch hydrogels formed at various crosslinking times revealed well-interconnected networks in all the hydrogel specimens. As the crosslinking time was extended from 2 to 24 h, the pore wall thickness and pore size of SN hydrogels increased. Quantitative analysis of the SEM micrographs was carried out and the results were summarized and showed in Table 1.

SEM images of SN starch hydrogels at various GA contents after 24 h of crosslinking time are shown in Figure 3. As the amount of GA in the hydrogels increased, the pore walls became thicker, and the pore size gradually rose. The pore wall thickness and pore size of these hydrogel were shown in Table 2. The observation was consistent with those reported by Bi Long et al. [36]. The average pore size of collagen type II/chitosan scaffold hydrogels increased with increasing the crosslinker concentration.

As can be seen in the SEM micrographs in Figure 2 and Figure 3 and Table 1 and Table 2, the average wall thickness and pore size of the SN starch hydrogels increased as the crosslinking time and GA content increased. As the crosslinking reaction was allowed to continue for a longer period of time or the concentration of GA was increased, the aldehyde groups of GA molecules were more likely to form additional crosslinks with the hydroxyl groups of starch molecules [37]. It should be noted that the pore number of SN starch hydrogels were decreased as a result of the increased wall thickness and pore size. Moreover, denser walls were observed when either the crosslinking time or the GA concentration was increased. This could be because the polymer chains became more interconnected [38]. These reasons could be deduced due to the lower overall free volume of the hydrogels, and consequently a decrease in water uptake percentage could be expected.

#### 3.2.2. Porosity of SN Starch Hydrogels

**Figure 4:**
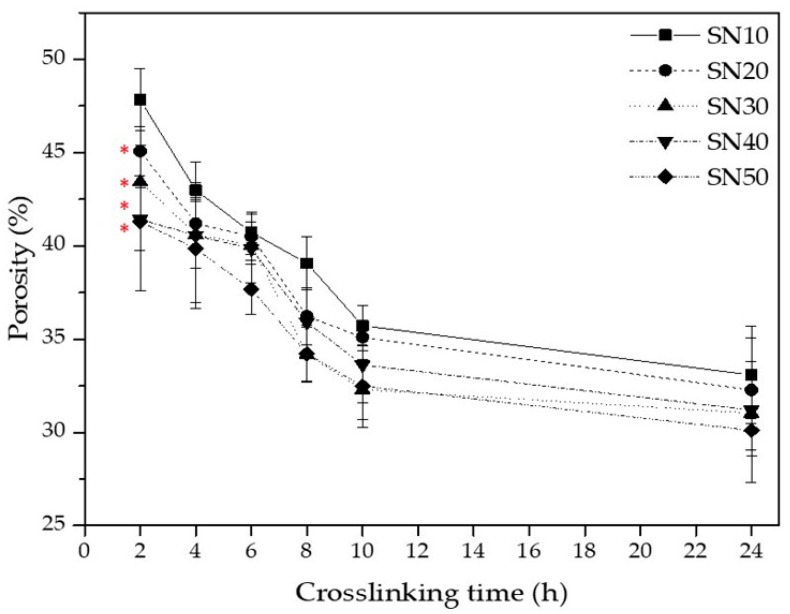
Porosity of SN starch hydrogels at various crosslinking times and GA contents (*n* = 3, * *p* < 0.05 compared with SN10).

The porosity of SN starch hydrogels prepared at various crosslinking times and GA contents was determined and illustrated in Figure 4. The porosity of SN starch hydrogels decreased linearly as the crosslinking time increased from 2–10 h. As the crosslinking time further increased up to 24 h, the decline levelled off. A similar trend was observed when the starch hydrogels were prepared at various GA contents. At 2 h of crosslinking, the porosity of the SN starch hydrogels significantly decreased as GA content rose. The maximum porosity of 47.83% and minimum porosity of 30.11% were obtained when the SN starch hydrogels were prepared with 10 GA for 2 h (SN10@2h) and with 50 mL of GA for 24 h (SN50@24h), respectively. This was plausibly due to the fact that when the crosslink density increased, the pore walls became thicker and denser. On the other hand, the pore count was significantly decreased with increasing crosslink density, thus lowering the overall free volume of the hydrogel (as shown in Figure 2 and Figure 3) [28,39].

#### 3.2.3. Water Uptake of SN Starch Hydrogels

**Figure 5:**
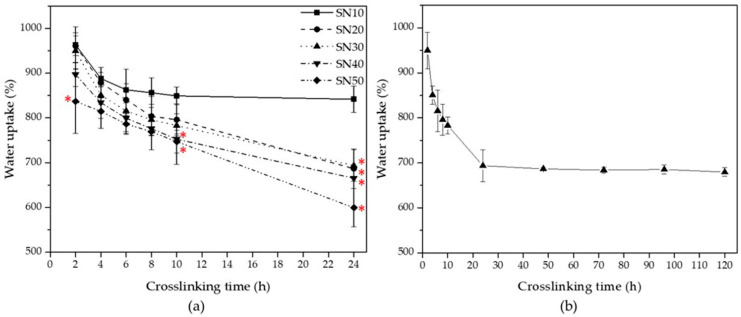
Water uptake of SN starch hydrogels; (**a**) various crosslinking times and GA contents (*n* = 3, * *p* < 0.05 compared with SN10) and (**b**) SN30 at various crosslinking times from 2–120 h.

The equilibrium water uptake of SN starch hydrogels at the different crosslinking times and GA contents are shown in Figure 5a. As crosslinking time and GA content increased, water uptake of the SN starch hydrogels decreased nonlinearly. The water uptake of SN starch hydrogel prepared with 10 mL GA declined rapidly from 2 to 4 h before leveling off and becoming constant. As stated earlier, water uptake was directly related to the degree of crosslinking of a hydrogel. This result could be due to the fact that the amount of GA used, 10 mL, yielded limited crosslinking degree and the reaction was completed in less than 6 h. The initial sharp decrease was also observed when the GA content increased to 20–50 mL. However, their water uptake percentage continued to decrease in a relatively linear fashion. Thus, Figure 5a) shown a continuing increase in crosslinking degree. To determine the optimal crosslinking time, starch SN30 hydrogels were prepared using crosslinking time 24–120 h. Figure 5b shows that the hydrogels’ water uptakes show little to no change after 24 h of crosslinking time. This finding revealed that 24 h was sufficient for the SN to completely and fully crosslink with the GA content used. Note that the higher degree of crosslinking also suggested a higher crosslink density of a hydrogel. Consequently, decreases in the free volumes as well as the mobility of polymer chains between the network were obtained [40]. These results were consistent with the reported porosities and morphologies of the hydrogels.

#### 3.2.4. Compressive Properties of SN Starch Hydrogels

**Figure 6:**
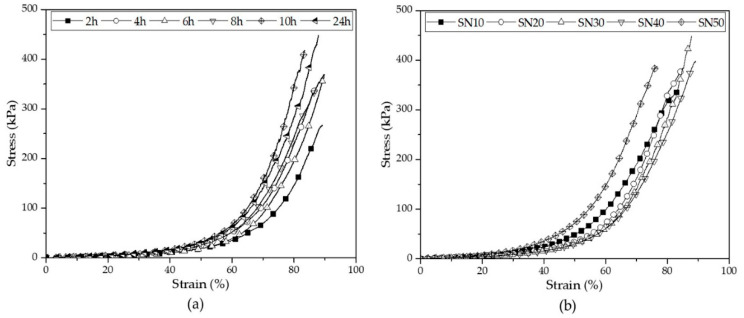
Stress-strain curves of SN starch hydrogels; (**a**) constant GA content at 30 mL with various crosslinking times (2–24 h) and (**b**) various GA contents (10–50 mL of GA) with constant crosslinking time at 24 h.

**Figure 7:**
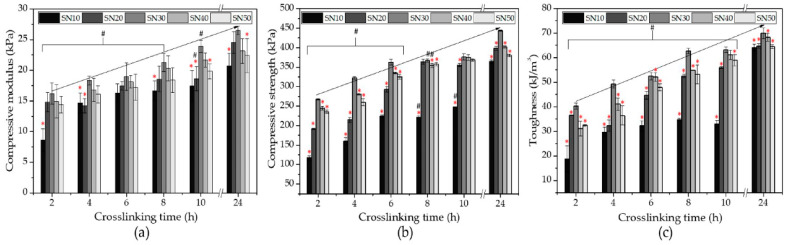
Compressive properties of SN starch hydrogels prepared with various crosslinker (GA) contents; (**a**) compressive modulus, (**b**) compressive strength and (**c**) toughness (*n* = 5, * *p* < 0.05 compared with SN30and ^#^ *p* < 0.05 compared with 24 h of crosslinking time).

Stress–strain curves of SN starch hydrogels at various crosslinking times and GA contents are shown in Figure 6a,b, respectively. From the stress–strain curves, it was found that all of the SN starch hydrogels were soft and flexible. To compare the effects of crosslinking time and GA contents of the mechanical properties of the SN hydrogels, their compressive strength, compressive modulus, and toughness were then plotted against crosslinking time, as illustrated in Figure 7.

As crosslinking time increased from 2 to 24 h, the compressive strength of the prepared starch hydrogels increased from 111.52 ± 5.26 kPa to 443.46 ± 5.76 kPa while their compressive modulus increased from 8.64 ± 1.84 kPa to 26.49 ± 0.69 kPa. Additionally, the increased crosslinking time increased the toughness of these hydrogels from 18.80 ± 5.23 kJ/m^3^ to 69.93 ± 2.17 kJ/m^3^. This indicated that increasing the crosslinking time allowed for a greater interaction between starch and GA molecules to occur.

At constant crosslinking time of 24 h, an increase in the GA content up to 30 mL resulted in increases of the compressive strength, compressive modulus, and toughness of the SN hydrogels, as shown in Figure 7. A further increase GA content of greater than 30 mL resulted in adverse decreases in the compressive properties of the SN hydrogels.

A similar observation was also reported by Bi Long et al. [36]. In their work, the compressive strength of collagen type II/chitosan scaffold hydrogels rose from 0.1 to 1.0 wt% genipin as a crosslinker. When genipin concentration was increased from 1.0 to 2.0 wt%, hydrogel compressive strength decreased. Their explanation was that the excessive use of genipin reduced crosslinking in the interior layers of the hydrogel, lowering its mechanical strength. Therefore, it is worth noting that, in some cases, increasing crosslinker content does not always result in an improvement in the mechanical properties of the hydrogel.

In our case, the mechanical strength of the hydrogels seemed to be largely dependent on their microstructure. Specifically, mechanical properties of a porous materials are largely influenced by the size of its pores, and smaller pores contribute to the enhancement of the mechanical strength of engineered structures [36,41]. Nonetheless, it is also well established that a higher degree of crosslinking (a thicker pore wall thickness in this case) can enhance the mechanical properties of the porous material [42]. The final hydrogel mechanical strength seemed to depend on the balance of these two effects.

In this study, 30 mL of GA was optimal content for preparing SN starch hydrogel with highest compressive properties (Figure 7). The hydrogel microstructure was responsible for the compressive property results. The SN starch hydrogels prepared using GA of less than 30 mL (SN10 and SN20) had thin pore walls and small pore sizes, which led to a poor structure support against compressive force. On the other hand, SN starch hydrogels containing GA of higher than 30 mL (SN40 and SN50) showed large pore sizes with thick and tall pore walls that collapsed easily. These pore structures led to a poor compression force distribution and thus structural fractures.

In summary, the important findings from the investigation of effects of crosslinking time and GA content on mechanical properties of SN starch-based hydrogels were that the optimal crosslinking time was 24 h and GA content was 30 mL. The final mechanical properties were mainly dependent on the microstructure of the porous hydrogels.

### 3.3. Characterization of Double Network (DN) Starch Hydrogels

To further enhance the mechanical properties of the starch-based hydrogel, the SN hydrogel with the highest compressive properties (SN30, 24 h crosslinking time) was chosen to be used as a primary network for fabricating the DN starch hydrogels. In this study, we developed DN starch hydrogels by introducing poly(vinyl alcohol) (PVA) as a secondary polymer and borax as a crosslinker. The PVA chains interpenetrated into the SN starch hydrogel network that served as the primary structure. The secondary network was then formed by the interaction of borate ions holding PVA strands together.

#### 3.3.1. Morphologies of DN Hydrogels

**Figure 8:**
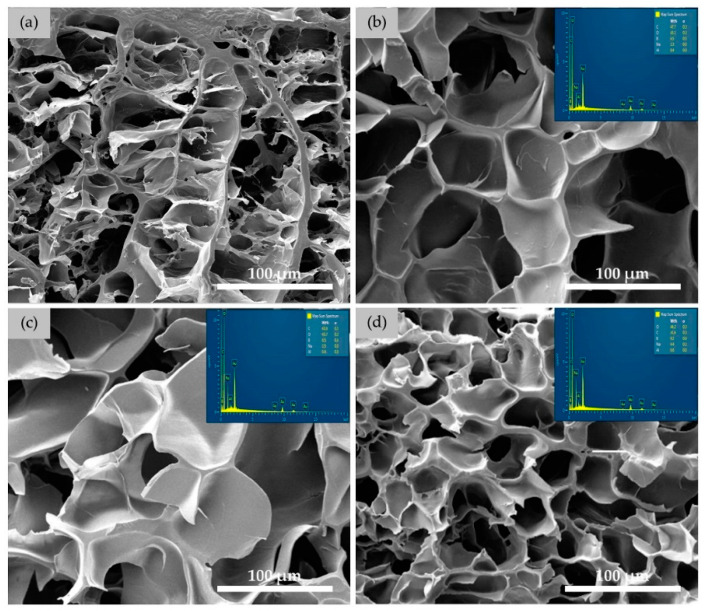
SEM micrographs and EDS spectra of DN starch hydrogels prepared with various borax concentrations (1000× of magnification): (**a**) SN30, (**b**) DN0.05, (**c**) DN0.10 and (**d**) DN0.15.

SEM micrographs and EDS spectra of SN30 and DN starch hydrogels prepared with various borax concentrations are shown in Figure 8. SEM micrographs showed that the porous structure of starch hydrogel changed with the presence of the second network. The major change occurred to the cell wall where they appeared smoother, denser, thicker, and more continuous. The pore size of the DN hydrogels was relatively larger than that of the SN hydrogel. The exception was found when the DN was prepared with borax at high concentration of 0.15 M where the pore distinctively appeared smaller than others DN hydrogels.

Table 3 summarizes the quantitative results from image analysis and Boron content evaluation. The presence of Boron atoms on the DN hydrogel surfaces was confirmed by EDS spectroscopy. Boron content increased linearly with increasing borax concentration used in the DN hydrogel preparation. The increased Boron content led to more borate ions, as required for the formation of a secondary network in DN hydrogels.

In Table 3, it can be observed that the pore wall thicknesses of the DN hydrogels increased with increasing borax concentration while the pore size of these hydrogels decreased. In comparison to the SN starch hydrogels, the DN starch hydrogels possessed three-dimensional structure with thicker walls and larger pores. These results suggested that the addition of PVA as a secondary network increased the pore wall thickness and pore size. The enlargement of the existing pore size may plausibly be explained by the penetration of a relative larger molecule of hydrated PVA into the starch hydrogel structure and thus expanded the pore to a greater degree. The affinity of water molecules with PVA molecules is due to the hydroxyl groups on the polymer chain [43]. The thicker wall was the result of the crosslinking reaction of penetrated PVA by borax, a secondary crosslinker, and formed the double network hydrogel. The overall crosslink density of the DN hydrogel was therefore higher than that of the SN hydrogel. Moreover, the increase in crosslink density took place when higher borax content was used, and at the highest borax content of 0.15 M, more crosslinking interactions [44] may lead to a more compact structure of the DN hydrogels [45]. The changes in the structure of the hydrogels were expected to further influence their compressive properties [46].

In Table 3, it can be observed that the pore wall thicknesses of the DN hydrogels increased with increasing borax concentration while the pore size of these hydrogels decreased. In comparison to the SN starch hydrogels, the DN starch hydrogels possessed three-dimensional structure with thicker walls and larger pores. These results suggested that the addition of PVA as a secondary network increased the pore wall thickness and pore size. The enlargement of the existing pore size may plausibly be explained by the penetration of a relative larger molecule of hydrated PVA into the starch hydrogel structure and thus expanded the pore to a greater degree. The affinity of water molecules with PVA molecules is due to the hydroxyl groups on the polymer chain [43]. The thicker wall was the result of the crosslinking reaction of penetrated PVA by borax, a secondary crosslinker, and formed the double network hydrogel. The overall crosslink density of the DN hydrogel was therefore higher than that of the SN hydrogel. Moreover, the increase in crosslink density took place when higher borax content was used, and at the highest borax content of 0.15 M, more crosslinking interactions [44] may lead to a more compact structure of the DN hydrogels [45]. The changes in the structure of the hydrogels were expected to further influence their compressive properties [46].

#### 3.3.2. Porosity of DN Starch Hydrogels

**Figure 9:**
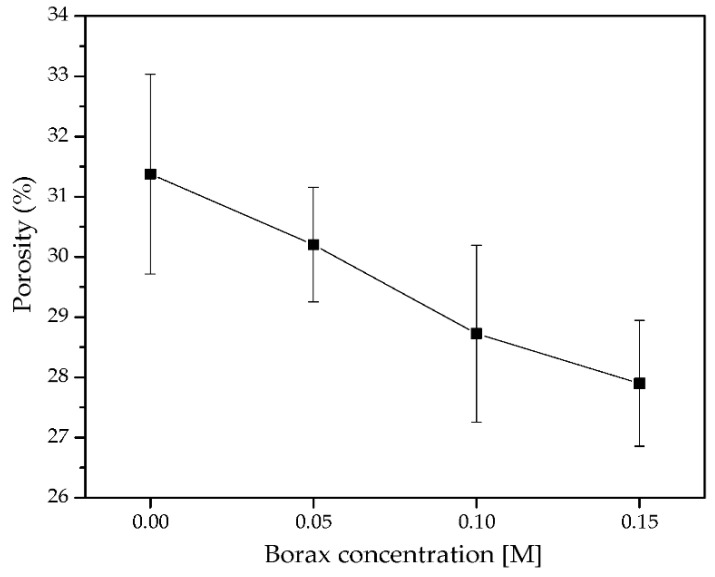
Porosity of DN starch hydrogels prepared at various borax concentrations (*n* = 3).

The porosity of SN hydrogel (SN30@24h) and its DN starch hydrogels prepared at various borax concentrations is shown in Figure 9. The graph shows the decreasing trend in the porosity of DN hydrogels as the borax concentration increased. The porosity of DN hydrogels was between 27% and 30%, which was lower than that of the SN hydrogel. These results well agreed with SEM micrographs (Figure 8) and the pore count shown in Table 3. A lower pore count and thicker wall could lead to a lower surface area and free volume. Thus, it was predictable that the porosity of DN hydrogels was lower than those of the SN hydrogels.

#### 3.3.3. Water Uptake of DN Starch Hydrogels

**Figure 10:**
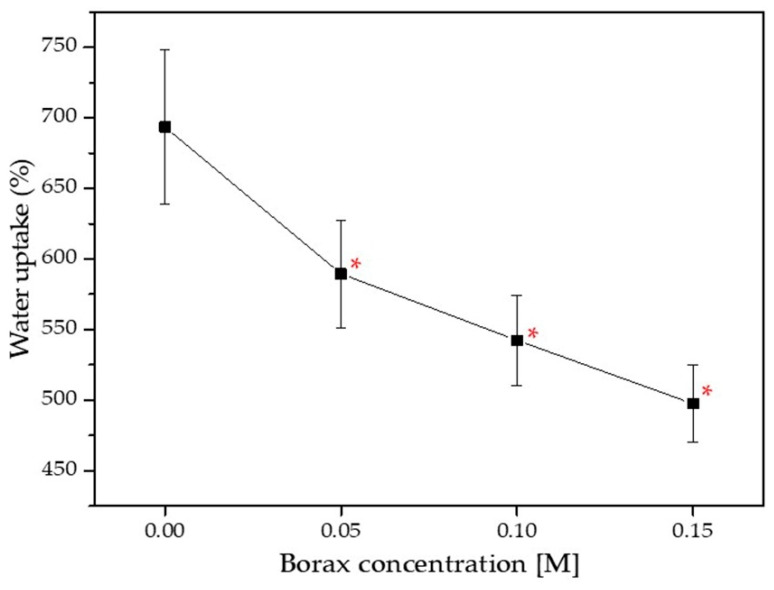
Water uptake of DN starch hydrogels prepared with various borax concentrations (*n* = 3, * *p* < 0.05 compared with SN30 hydrogel (0.00 M of borax concentration).

After 24 h of immersion in deionized water, water uptake percentages of the DN hydrogels were determined. It was found that the water uptake of the DN was linearly dependent on the amount of borax concentration used. The water uptake capacity of DN hydrogels significantly decreased with increasing borax concentration (* *p* < 0.05, compared with SN30). Moreover, the DN0.15 hydrogel had the lowest water uptake, measuring at 497.58%, as shown in Figure 10. Two possible causes may contribute to the decrease of water uptake as the crosslinker concentration increased. The first cause was directly related to the increase in the crosslinking density. Free volume in a hydrogel is generally decreased as a crosslinking density increases. The second cause was due to a reduction in the number of hydrophilic groups. As a result of the reduction in the hydrophilic segment of the DN hydrogel, it became more difficult for water molecules to interpenetrate into the structure of the hydrogel, which led to a reduction in the water uptake capacity of the DN hydrogel [44].

#### 3.3.4. Compressive Properties of DN Starch Hydrogels

**Figure 11:**
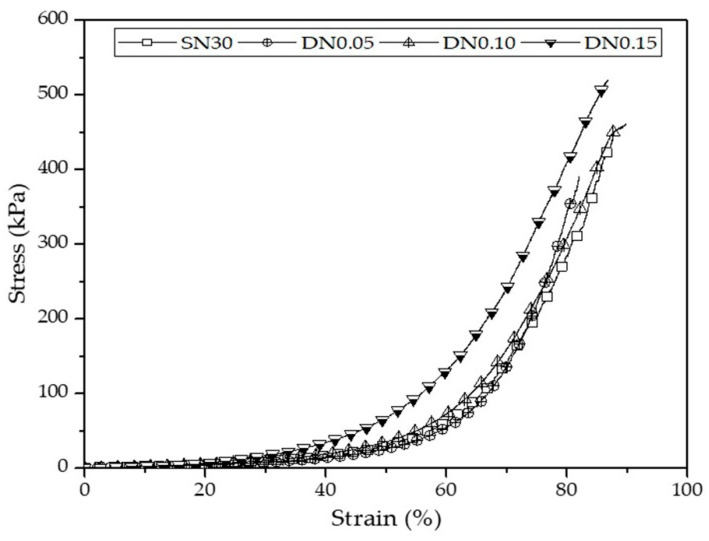
Stress-strain curves of SN30 and DN starch hydrogels prepared with various borax concentrations.

Stress–strain curves of DN hydrogels are shown in Figure 11. The values of compressive stress and compressive modulus of the DN hydrogels are summarized in Table 4. As expected, the compressive properties of DN hydrogels were superior to those of SN starch hydrogels. The addition of PVA and borax increased the chain density in the DN hydrogels while also increasing chain interaction [46]. At 0.15 M of borax, the compressive strength, compressive modulus, and toughness of the DN hydrogels were significantly higher than other groups, * *p* < 0.05, compared with DN0.15. Among these, the DN0.15 exhibited the highest compressive strength (496.73 ± 20.80 kPa), compressive modulus (33.99 ± 2.71 kPa), and toughness (90.53 ± 3.38 kJ/m3). The results revealed that the presence of the secondary network in the DN hydrogel increased polymer chain entanglement and crosslink density, reducing chain freedom in comparison to a single network hydrogel [12,47,48]. In addition, the compressive modulus of the DN hydrogels ranged from 25 to 35 kPa, which was comparable to the compressive modulus of articular cartilage in the superficial zone (20–1160 kPa) [22]. The results indicated that these DN hydrogels possessed an adequate compressive modulus for use in articular cartilage replacement.

### 3.4. In Vitro Cytotoxicity

**Figure 12:**
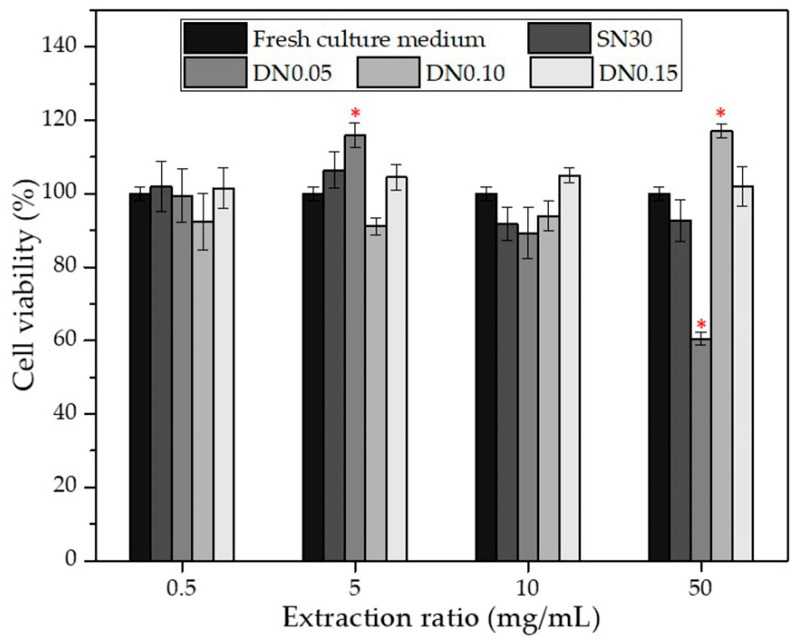
Viability of human chondrocytes after incubating with hydrogels extracted culture media at different extraction ratio (*n* = 3, * *p* < 0.05 compared with fresh culture medium).

The MTT cell cytotoxicity assay is one of the in vitro screening tests used to evaluate the effects of materials on cellular growth at various extraction ratios. In this study, chondrocyte cell viability in the hydrogel extractions was investigated. The hydrogel extractions at concentrations of 0.5, 5, 10, and 50 mg/mL were compared against a fresh culture media. Figure 12 shows the cell viability after 24 h treated with extractions of various types of hydrogels. The cell viability of all but one condition was in the range of ~89~116%. The results indicated that the hydrogel samples were non-toxic to chondrocytes, as defined by ISO 10993-5 (cell viability ≥ 70%). The only exception was the result of 50 mg/mL of extract medium of DN0.05 hydrogel where the cell viability was ~60%. The plausible explanation was that the low borax concentration of 0.05 M was insufficient to form a stable network structure. Thus, the DN0.05 hydrogel degraded more rapidly as compared to the hydrogels prepared with higher borax contents. The higher concentration of the hydrogel extract could contain a greater amount of disassociated borax and cause cell apoptosis [49]. According to our findings, the SN30, DN0.10, and DN0.15 hydrogels have potential for tissue engineering applications.

## 4. Conclusions

Starch hydrogels have been proven to be promising candidates for usage in medical applications. Their limitations, however, are generally due to poor mechanical properties, which need to be addressed before their efficacy can be realized. We have demonstrated in this study that the mechanical properties of starch hydrogels could be improved by using a double network strategy. We synthesized crosslinked double-network hydrogels where starch was a primary network and poly(vinyl alcohol) was a secondary network. We reported the parameters affecting the hydrogels’ properties. For SN starch hydrogel, the compressive properties increased with increasing crosslinking time. We found the optimal content of the primary crosslinker (GA) content in SN starch hydrogel to be 30 mL, the SN30, which gave the highest compressive properties. The SN starch hydrogel prepared with the optimal condition was further improved by synthesizing into DN hydrogels. The compressive properties of DN hydrogels increased with increasing borax (the secondary crosslinker) concentration. The DN hydrogel prepared with 0.15 M of the borax solution exhibited the highest compressive properties. The values were higher than those of the SN starch hydrogels. The mechanical properties, porosity, and water uptake capability of the hydrogels were all well related to their microstructures. In addition, an in vitro cytotoxicity test revealed that the stable DN hydrogels were safe to chondrocytes. The optimal crosslinker concentration and crosslinking time are essential parameters that influence the final properties of the hydrogel. It is crucially important to systematically determine these parameters in order to achieve a hydrogel with the desired properties. This study successfully proved that a double networking strategy could be used as a tool to improve the mechanical properties of starch-based hydrogels. Further improvements are required to obtain hydrogels with properties suitable for tissue engineering applications. Our ongoing research focuses on the fabrication of tougher hydrogels using combined strategies, i.e., double network together with nanocomposite. The preliminary results show the promising development of hydrogels with improved mechanical properties and enhanced osteoblast cell growth.

## Figures and Tables

**Table 1:** Effect of crosslinking time on wall thickness, pore size, and pore count of SN hydrogels prepared with a constant GA content of 30 mL and various crosslinking times.

Sample	Crosslink-Time (h)	Wall Thickness (µm)	Pore Size (µm)	Pore Count (n */100 µm^2^)
SN30	2	0.79 ± 0.06	5.85 ± 0.08	166.75 ± 8.30
SN30	4	1.54 ± 0.21	13.51 ± 0.28	106.33 ± 8.49
SN30	6	2.00 ± 0.31	18.49 ± 0.68	31.33 ± 5.35
SN30	8	2.81 ± 0.18	18.70 ± 0.23	28.25 ± 5.30
SN30	10	3.32 ± 0.16	19.97 ± 0.34	26.25 ± 1.89
SN30	24	3.77 ± 0.81	23.15 ± 0.47	20.75 ± 2.79

* n is number of pore presence in a defined area.

**Table 2:** Effect of GA content on wall thickness, pore size, and pore count of SN hydrogels prepared using a constant crosslinking time of 24 h and various GA contents.

Sample	GA Content (mL)	Wall Thickness (µm)	Pore Size (µm)	Pore Count (n/100 µm^2^)
SN10	10	1.51 ± 0.26	16.27 ± 0.38	25.33 ± 4.79
SN20	20	2.07 ± 0.27	17.08 ± 0.22	23.75 ± 5.01
SN30	30	3.77 ± 0.81	23.15 ± 0.47	20.75 ± 2.79
SN40	40	3.91 ± 0.33	23.35 ± 0.93	18.25 ± 1.71
SN50	50	5.16 ± 0.50	26.68 ± 0.63	16.50 ± 3.00

**Table 3:** Effect of borax concentration on wall thickness, pore size, and pore count of DN hydrogels.

Sample	Boron Content * (%)	Wall Thickness (µm)	Pore Size (µm)	Pore Count (n/100 µm^2^)
SN30	-	3.77 ± 0.81	23.15 ± 0.47	20.75 ± 2.79
DN0.05	6.57 ± 0.31	5.86 ± 0.72	55.18 ± 0.77	2.49 ± 0.61
DN0.10	8.73 ± 0.49	7.11 ± 0.66	53.55 ± 0.34	3.33 ± 0.58
DN0.15	9.70 ± 0.50	8.04 ± 0.64	23.31 ± 0.72	9.00 ± 1.63

* Boron content was estimated by EDS spectrum.

**Table 4:** Compressive properties of SN starch hydrogel (30 mL of GA and 24 h of crosslinking time) and DN starch hydrogels (*n* = 5, * *p* < 0.05 compared with DN0.15 hydrogel).

Sample	Compressive Strength at Break (kPa)	Compressive Modulus (at 5–10% Strain) (kPa)	Toughness (kJ/m^3^)
SN30	443.46 ± 11.76 *	26.49 ± 0.69 *	69.93 ± 2.17 *
DN0.05	429.50 ± 20.84 *	26.85 ± 1.76 *	77.77 ± 0.62 *
DN0.10	462.83 ± 18.18 *	28.12 ± 2.88 *	80.03 ± 1.75 *
DN0.15	496.73 ± 20.80	33.99 ± 2.71	90.53 ± 3.38

## Data Availability

The corresponding author can provide the data that were used to support the study’s conclusions upon request.
